# Training Community African American and Hispanic/Latino/a Advocates on Prostate Cancer (PCa): a Multicultural and Bicoastal Approach

**DOI:** 10.1007/s13187-023-02326-4

**Published:** 2023-07-14

**Authors:** Carolina Aristizabal, Sandra Suther, Yingwei Yao, Linda S. Behar-Horenstein, Fern Webb, Mariana C. Stern, Lourdes Baezconde-Garbanati

**Affiliations:** 1 Department of Preventive Medicine, Keck School of Medicine, University of Southern California, Los Angeles, USA; 2 Norris Comprehensive Cancer Center, University of Southern California, Los Angeles, CA USA; 3 College of Pharmacy & Pharmaceutical Sciences, Institute of Public Health, Florida Mechanical and Agricultural University (FAMU), Tallahassee, USA; 4 College of Medicine, Department of Surgery, University of Florida (UF), Gainesville, USA

**Keywords:** African American, Hispanic/Latino/a, Public health education, Cancer advocates, Prostate cancer

## Abstract

African American communities are disproportionately impacted by prostate cancer (PCa) compared to other racial/ethnic groups. Whereas the incidence of PCa in Hispanic/Latino men is lower than the incidence in non-Hispanic/Latino White men, Hispanic/Latino men are more likely to be diagnosed with PCa in late stages, and less likely to be knowledgeable about PCa, resulting in significant disparities. We developed, culturally adapted, translated, implemented, and evaluated a PCa Cancer Advocacy Training in African American and Hispanic/Latino/a communities. Culturally and language specific content for African American and Hispanic/Latino/a patients on PCa causes, risk factors, epidemiology, detection, diagnosis, and treatment were delivered through a workshop and simultaneously broadcasted in Spanish in Los Angeles County (*n* = 29) and in English in Tallahassee, FL (*n* = 9). Pre- and posttest surveys assessed impact. Pre vs post differences were statistically significant in knowledge (5.0 ± 1.6 vs 6.3 ± 1.1) and advocacy intentions (3.9 ± 0.9 vs 4.3 ± 0.8), on correctly identifying warning signs for PCa (50% vs 87%), intent to inform and educate about PCa within the next 3 months (69% vs 95%), to ensure that high-quality research is sensitive to the priorities of patients (63% vs 84%), to help increase patient recruitment, compliance, and retention for clinical trials within the next month (62% vs 84%), intent to engage in PCa patient education within the next 3 months (67% vs 92%), and in engaging in PCa community outreach within the next 3 months (67% vs 94%). There were no significant differences due to race/ethnicity. The Cancer Advocacy Training led to increased knowledge, awareness, and intention to engage in advocacy regarding PCa in the next 3 months. Results suggest that delivering culturally and language specific educational information increases engagement of Hispanic/Latino/a and African American patient/community advocates.

## Introduction

Prostate cancer (PCa) is the second most common cancer among men worldwide, after lung cancer, with an estimated 1.2 million men diagnosed in 2018 [1–3]. Compared to other cancers, the incidence and prevalence of PCa significantly increases as men age, with an average age of 66 at diagnosis [4]. Data from 2019 show that nearly 20% of men are diagnosed with prostate cancer at some point during their lifetime, totaling 3,253,416 men living with PCa in the USA. Regarding mortality, more men die from prostate cancer in the USA compared to other cancers [5]. In 2019, the overall death rate was 18.8 per 100,000 men per year [5]. In addition, PCa incidence and mortality significantly differ by race and ethnicity with incidence rates being highest among African American, followed by Non-Hispanic/Latino White, Hispanic/Latino, Asian, and American Indian/Alaskan Native men [5, 6].

Evidence shows that detecting PCa after it has progressed to organs (metastasis) significantly limits survival [7]. However, if diagnosed and detected early, PCa can be treated as evidenced by a 5-year relative survival of 97.8% [8]. The digital rectal exam (DRE) and the prostate specific antigen (PSA) test are two conventional methods for the early detection of PCa [9]. Evidence shows that adopting prevention measures and screening reduces mortality rates for PCa, and prognosis can be optimized [10]. Studies have shown that men who have higher levels of PCa knowledge are more likely to screen [10–12]. To ensure the dissemination of timely and accurate information regarding screening and effective treatment, it is critically important to implement strategies for public health education in the community, particularly among those who suffer from this disease at disproportionately higher rates [13].

Identifying effective strategies and approaches to increase men’s knowledge about and motivation to be screened and treated for PCa can be challenging. Studies have shown that men are less concerned about their health than women, to the point of disregarding warning signs of disease [14]. For example, men tend to have fewer medical appointments than women, and also tend to adopt more unhealthy lifestyle practices compared to women [15]. African American and Hispanic/Latino men, in particular, have several individual and societal challenges that further reduce their adherence to PCa screening and timely access to treatment [16]. Both African American and Hispanic/Latino men in the USA are reported to have lower PSA screening rates than non-Hispanic/Latino White men [17, 18]. Societal barriers such as a lack of health insurance, lack of awareness about the importance of PCa screening, lack of health literacy, language barriers among immigrant populations, fears, and cultural values and behaviors may contribute to lower rates of screening and disparities in early detection and treatment among Hispanic/Latino and African American men [19]. Men who have higher levels of knowledge about PCa are more likely to be screened [10, 20] and subsequently treated. Therefore, it is essential to develop and implement programs that provide accurate and culturally tailored information aimed at reducing barriers and increasing screening among African American and Hispanic/Latino men in our communities. To be successful, these programs need to include cultural and language specific content, address misconceptions and barriers specific to those communities, and be delivered through communication methods that are acceptable in the target communities [21].

Underserved ethnically disparate populations benefit from heath education outreach efforts when they are conveyed and translated by specially trained peers from the respective cultural enclaves. Individuals who have the respect and trust of the community and live in the community are better equipped to understand the unique cultural needs of their communities and are more qualified to translate the mission of comprehensive health care to community peers than non-community members. Researchers have shown that when community residents are trained as health advisors/ambassadors, the community is more likely to view them as caring, credible, and knowledgeable advice givers. Trained residents within the community typically understand the beliefs, attitudes, and behaviors of the informal social groups that historically exist in the community. This cultural knowledge and social consciousness empower community health advisors to be viewed as trustworthy in offering advice [22]. Studies have shown that community health workers and educators, also known as “*promotores de salud*” (Hispanic/Latino/a community health workers), and patient advocates are effective in delivering cancer education information [23, 24].

To reduce disparities in PCa knowledge, awareness, screening, and access to treatment among African American and Hispanic/Latino/a populations, the Florida-California Cancer Research Education and Engagement (CaRE^2^) Health Equity Center Community Outreach Core (COC) developed and evaluated a training program specifically designed to engage African American and Hispanic/Latino/a community health workers, *promotores de salud*, and PCa patient advocates, to raise PCa awareness while disseminating PCa information in a culturally grounded and language specific manner. We report the efficacy of this training program that was designed to increase knowledge, awareness, and advocacy intentions of community health worker and patient advocate participants.

## Methods

### Description of Program

The cancer advocacy training was designed to be conducted in person utilizing the *Handbook for Prostate Cancer Advocacy: Principles & Best Practices* [25]. The handbook was developed and tailored to the African American community by our partners at the University of Florida. Using a similar approach, we developed a PCa manual (toolkit) “Raising Awareness on Prevention of Prostate Cancer in Latino Men” by tailoring it to the Hispanic/Latino community. This handbook was designed and adapted in Spanish and English. Both handbooks contain information about the prostate, its location, prostate health, what prostate cancer is, its risk factors, screening, incidence, and mortality rates specific for each community, treatment, and survivorship. The goal of this manual is to provide advice, strategies, data, and talking points to raise awareness on prostate cancer and to encourage African American and Hispanic/Latino men to be screened.

The training workshop was conducted simultaneously at the University of Southern California (USC) Norris Comprehensive Cancer Center in Los Angeles, CA, and the Florida Agricultural and Mechanical University (FAMU) in Tallahassee, FL, in both English and Spanish. Culturally and language specific content on PCa cause, risk factors, epidemiology, detection, diagnosis, and treatment were reviewed using a score card (Materials Review Score card) which is a tool developed by TEAM Lab at USC to systematically evaluate materials based on important factors that make a material suitable for the intended audience. This tool is useful to identify gaps and weaknesses in materials and provide guidance on what areas of the material need to be improved. Training was delivered utilizing these materials by CaRE^2^ research scientists and broadcasted through interactive and live webinars in Spanish in Los Angeles and in English in Tallahassee. These cities represent the catchment areas of our CaRE^2^ partnership institutions, as well as two regions with very high proportion of Hispanic/Latino (Los Angeles) and African American men (Tallahassee). The sessions consisted of a plenary session for each group, five talks by CaRE^2^ scientists and clinicians, and two break-out sessions. Participants were able to choose which session or webinar (Spanish or English language) they wanted to attend. All participants were provided a toolbox that included the toolkit for either Hispanic/Latino or African American men, educational materials such as brochures from the American Cancer Society regarding PCa and brochures regarding participation in cancer research and clinical trials, as well as resource directory to refer community members to ongoing studies and additional information. A PCa book in Spanish titled “*No le tenga miedo al dedo*”; a prostate shaped anti-stress ball; a USB with a video from Urologist Dr. Rene Sotelo explaining what is PCa, its signs, symptoms, screening, and treatment; a notepad; and a pen were provided to the Los Angeles participants. An experienced cancer advocate provided guidance on how to use the materials in the prostate cancer toolbox. All participants completed an informed consent and were invited to take the pre- and posttest training surveys.

### Training Surveys/Evaluation Tools

The pretest included demographic questions (race/ethnicity, educational attainment, employment status, zip code) and general questions regarding PCa (its cause, screening, warning signs, diagnosis) as well as questions assessing beliefs about PCa, expectations for the PCa advocacy training, and intentions to advocate at the community and policy levels. The posttest consisted of the same type of questions but without demographics. Pretest and posttest questions designed to assess participant knowledge and beliefs about PCA were adapted from the Global Prostate Cancer Measures (Transatlantic Consortium (CaPTC)) [26] and the African Caribbean Cancer Consortium (AC3) CaPTC-AC3 [27]. Items related to participant advocacy beliefs were adapted from CaPCaS Phase II Advocacy Survey [26, 27]. Some items related to participant beliefs about the effectiveness of the Cancer Advocacy Training Workshop were developed by the Planning and Evaluation group (PEC) from CaRE^2^ while other items were adapted from the *International Workshop on Cancer Advocacy for African Countries* (CAAC) [28]. Multiple choice, Likert scales, and dichotomous questions were included. The surveys were administered in person in Los Angeles and Tallahassee during the training workshop, after the opening remarks, prior to the first session, and at the end of the last session before the closing remarks.

### Data Analysis

Data were imported into statistical software R (version 3.5.1) for analysis. Descriptive statistics of demographics and outcome measures were calculated. A comparison of pretests and posttests was conducted to assess impact of the PCa workshop. The Benjamini-Hochberg (BH) method [29] was used to adjust for multiple testing. An adjusted *p* < 0.05 was considered statistically significant.

## Results

A total of 38 participants completed the pretest, the training, and the posttest in Los Angeles (*n* = 29) and in Tallahassee, FL (*n* = 9) (Table 1). Most participants self-reported as Hispanic/Latino (55%). In terms of race, 32% self-reported as White and 26% self-reported as Black or African American. About a third (32%) completed only high school or less while 37% completed college and 26% completed graduate school (Table 1).

**Table 1:** Participant demographics

Variable	Category	*n* (%)
1. Location	CA	29 (76%)
	FL	9 (24%)
2. Race	White	12 (32%)
	Black	10 (26%)
	Other	9 (24%)
	Unknown	7 (18%)
3. Ethnicity	Hispanic/Latino/a	21 (55%)
	Non-Hispanic/Latino/a	11 (29%)
	Unknown	6 (16%)
4. Education	High school or less	12 (32%)
	College/post-secondary	14 (37%)
	Graduate/professional	10 (26%)
	Unknown	2 (5%)

### Pre- and Posttest Scores

Significant pre- to post-training improvements were observed in knowledge (5.0 ± 1.6 vs 6.3 ± 1.1, *p* < 0.001, Table 2). We also observed higher advocacy intentions (3.9 ± 0.9 vs 4.3 ± 0.8, *p* < 0.001) at posttest (Table 2). Adjusting for baseline scores, there were no significant differences on knowledge and advocacy scores by race/ethnicity, location, or educational level (Table 3). The percentages of correct answers at posttest were higher for all items (items #1 to #8, Table 4) than at pretest, with the improvement on identifying warning signs for PCa (item #3) being statistically significant (50% vs 87%, *p* = 0.02). The lowest percent of accurate responses was for screening age (item #2) with only 13% answering correctly at pretest and 18% at posttest. Significant pre- to posttest improvements were observed in seven advocacy items (Table 5), including intent to inform and educate patients (item # 3), family, and friends about PCa within the next 3 months (*p* = 0.01), to ensure that high-quality research is sensitive to the priorities of patients (item #5) (*p* = 0.01), to help increase patient recruitment, compliance, and retention for clinical trials within the next month (item #6) (*p* = 0.01), intent to engage in PCa research (item #8) (*p* = 0.04), support services (item #9) (*p* = 0.04), patient education (item #10) (*p* = 0.04) within the next 3 months, and in engaging in PCa community outreach within the next 3 months (item #11) (*p* = 0.01) (Table 5). The participants had strong expectations about the workshop. At pretest, 100% expected to receive information on PCa cancer research and resources for use in community level education. At posttest, 97% reported that they received the information from the workshop.

**Table 2:** Pretest and posttest mean (standard deviation) scores in scales^a^

Variable	Pretest	Posttest	*p*
Knowledge	5.0 (1.6)	6.3 (1.1)	<0.001
Advocacy	3.9 (0.9)	4.3 (0.8)	<0.001

^a^Denotes when applicable compares pretest and posttest scores

**Table 3:** Knowledge scores by education level, ethnicity, and race

Outcome	Variable	Category	Pretest	Posttest	Diff	*p*
Knowledge	Education	High school or less	4.1 (1.8)	5.9 (1.3)	1.8 (2.1)	0.80
		College/post-secondary	5.1 (1.2)	6.4 (1.0)	1.3 (1.4)	
		Graduate/professional	5.7 (1.8)	6.3 (0.9)	0.6 (1.5)	
	Ethnicity	Hispanic/Latino/a	4.4 (1.8)	6.0 (1.2)	1.6 (2.0)	0.70
		Non-Hispanic/Latino/a	5.6 (1.3)	6.5 (0.7)	0.8 (1.3)	
	Race	White	5.2 (1.1)	6.2 (1.2)	0.9 (1.4)	0.70
		Black	5.7 (1.2)	6.6 (0.8)	0.9 (1.1)	
		Other	4.0 (2.3)	5.7 (1.2)	1.7 (2.3)	
	Location	CA	4.7 (1.7)	6.0 (1.1)	1.3 (1.8)	0.18
		FL	6.1 (0.9)	7.1 (0.6)	1.0 (1.0)	
Advocacy	Education	High school or less	3.9 (0.9)	4.2 (1.0)	0.4 (0.8)	0.72
		College/post-secondary	3.6 (1.0)	4.2 (0.7)	0.6 (0.6)	
		Graduate/professional	4.1 (0.6)	4.5 (0.6)	0.4 (0.5)	
	Ethnicity	Hispanic/Latino/a	3.8 (0.8)	4.3 (0.9)	0.5 (0.7)	0.70
		Non-Hispanic/Latino/a	4.0 (1.2)	4.3 (0.8)	0.3 (0.6)	
	Race	White	3.9 (1.0)	4.1 (1.0)	0.2 (0.6)	0.70
		Black	4.0 (1.1)	4.3 (0.7)	0.3 (0.6)	
		Other	3.6 (0.8)	4.3 (0.7)	0.7 (0.6)	
	Location	CA	3.8 (0.8)	4.3 (0.8)	0.5 (0.6)	0.70
		FL	4.0 (1.1)	4.3 (0.7)	0.3 (0.6)

**Table 4:** Statistics for knowledge test items

Item	Pretest	Posttest	*p*
1. Causes	79%	89%	0.62
2. Screening age	13%	18%	0.88
3. Signs (which of the following is a warning sign of prostate cancer?)	50%	87%	0.02
4. Type of screening	50%	61%	0.67
5. Men over the age of 40 should talk to their doctor about their risk for prostate cancer	89%	92%	1
6. Having somebody in your family with prostate cancer increases the chance of getting prostate cancer	76%	95%	0.19
7. The chance of getting prostate cancer goes up with age	74%	97%	0.06
8. A diet high in fat will decrease the chance of getting prostate cancer	68%	87%	0.29

**Table 5:** Statistics for advocacy and workshop quality items

Item	Category	Pretest	Posttest	*p*
1. I will actively work and speak out for the benefit of others in my communities about cancer within the next 3 months.	Strongly disagree	3%	0%	0.10
	Disagree	0%	3%	
	Neutral	13%	5%	
	Agree	37%	24%	
	Strongly agree	47%	68%	
2. I will actively work and speak out for the benefit of others in my communities about prostate cancer within the next 3 months.	Strongly disagree	3%	0%	0.17
	Disagree	0%	3%	
	Neutral	18%	5%	
	Agree	29%	29%	
	Strongly agree	50%	63%	
3. I will inform and educate cancer patients, family members, and friends about prostate cancer within the next 3 months.	Strongly disagree	3%	0%	0.01*
	Disagree	5%	3%	
	Neutral	24%	3%	
	Agree	32%	32%	
	Strongly agree	37%	63%	
4. I will seek to impact public policy through lobbying at the local, state, and/or federal level about prostate cancer within the next 3 months.	Strongly disagree	0%	0%	0.89
	Disagree	8%	8%	
	Neutral	21%	22%	
	Agree	34%	32%	
	Strongly agree	37%	38%	
5. I will work to ensure that high-quality research is sensitive to the priorities of cancer patients within the next 3 months.	Strongly disagree	3%	3%	0.01*
	Disagree	11%	0%	
	Neutral	24%	14%	
	Agree	22%	30%	
	Strongly agree	41%	54%	
6. I will assist with strategies to increase patient recruitment, compliance, and retention for clinical trials within the next month.	Strongly disagree	5%	3%	0.01*
	Disagree	14%	5%	
	Neutral	19%	8%	
	Agree	32%	34%	
	Strongly agree	30%	50%	
7. I will provide support to cancer patients and their families within the next 3 months.	Strongly disagree	5%	3%	0.07
	Disagree	8%	3%	
	Neutral	18%	13%	
	Agree	42%	32%	
	Strongly agree	26%	50%	
8. I will engage in prostate cancer research within the next month.	Strongly disagree	8%	3%	0.04*
	Disagree	11%	11%	
	Neutral	27%	8%	
	Agree	22%	29%	
	Strongly agree	32%	50%	
9. I will engage in prostate cancer support services, within the next 3 months.	Strongly disagree	11%	3%	0.04*
	Disagree	11%	5%	
	Neutral	22%	8%	
	Agree	24%	39%	
	Strongly agree	32%	45%	
10. I will engage in prostate cancer patient education within the next 3 months.	Strongly disagree	8%	3%	0.04*
	Disagree	8%	0%	
	Neutral	16%	5%	
	Agree	24%	46%	
	Strongly agree	43%	46%	
11. I will engage in prostate cancer community outreach within the next 3 months.	Strongly disagree	6%	3%	0.01*
	Disagree	8%	0%	
	Neutral	19%	3%	
	Agree	25%	35%	
	Strongly agree	42%	59%	
12. In general, I would rate the speakers as outstanding.	Strongly disagree		0%	
	Disagree		0%	
	Neutral		0%	
	Agree		24%	
	Strongly agree		76%	
13. I found the advocacy toolkit/toolbox to be a great resource and will use it in my practice.	Strongly disagree		0%	
	Disagree		0%	
	Neutral		5%	
	Agree		16%	
	Strongly agree		79%	
14. The program material was presented at an appropriate level	Strongly disagree		0%	
	Disagree		0%	
	Neutral		0%	
	Agree		24%	
	Strongly agree		76%	
15. The program content was objective, balance, and free from commercial bias or influence.	Strongly disagree		3%	
	Disagree		0%	
	Neutral		0%	
	Agree		19%	
	Strongly agree		78%	
16. The program met my expectation in accomplishing the stated educational objectives.	Strongly disagree		0%	
	Disagree		0%	
	Neutral		0%	
	Agree		19%	
	Strongly agree		81%	
17. Overall, the quality of rating of the Prostate Cancer Advocacy Training Workshop Program was excellent.	Strongly disagree		0%	
	Disagree		0%	
	Neutral		0%	
	Agree		16%	
	Strongly agree		84%	
18. The content of presentations	Poor		0%	
	Fair		0%	
	Good		11%	
	Very good		11%	
	Excellent		79%	
19. Relevance of presentations and discussions	Poor		0%	
	Fair		0%	
	Good		5%	
	Very good		18%	
	Excellent		76%	
20. The presentation delivery	Poor		0%	
	Fair		3%	
	Good		5%	
	Very good		22%	
	Excellent		70%	
21. Response to questions	Poor		0%	
	Fair		0%	
	Good		0%	
	Very good		32%	
	Excellent		68%	
22. Visual aids and handouts	Poor		0%	
	Fair		3%	
	Good		3%	
	Very good		16%	
	Excellent		79%	
23. Teaching methods	Poor		0%	
	Fair		0%	
	Good		5%	
	Very good		29%	
	Excellent		66%	

*Denotes statistical significance, *p* < 0.05

## Discussion

Through our developed Prostate Cancer Advocacy Training, we aimed to provide community health workers and patient advocates with tools and strategies that would improve their ability to effectively share PCa information with African American and Hispanic/Latino/a populations. Unique features of our training program included: (1) the use of culturally tailored and adapted content, provided by cancer experts in lay language for African American and Hispanic/Latino/a communities, (2) inclusion of African American and Hispanic/Latino/a scientists and trainers, (3) simultaneous training in Spanish and English, with live broadcasting between Tallahassee, FL and Los Angeles, CA, in participants’ preferred languages. Training was successful in increasing participants’ knowledge of PCa, as well was their awareness and intentions to advocate.

Knowledge about various PCa aspects before the training varied widely by topic, with knowledge about screening age (13%), types of screening (50%), and signs of PCa (50%) being the ones with lowest knowledge. A previous study targeting African American men conducted in Tallahassee, FL, showed that 83% of participants presented some knowledge on PCa screening, while 17% had no knowledge [30]. Similarly, in another study, African American men residing in New York reported an awareness of PCa screening at a rate of 57% [31]. Therefore, our findings show lower levels of knowledge about aspects of PCa screening in our participants compared to other studies. These findings suggest that it is essential to develop and implement culturally responsive health education interventions tailored to the community’s level of knowledge. Findings from our study concur with previous studies in which participants showed a substantial increase in knowledge level following prostate cancer interventions [32, 33]. However, they also suggest that education about specific aspects of PCa screening through our training, such as recommended age, needs to be improved as knowledge gains after training were still modest.

Besides increasing PCa knowledge and awareness among these populations, challenges remained driven by hesitancy of some men to talk with their primary care physicians/health providers about their risk of developing PCa and the benefits of screening. Men from vulnerable populations often distrust health care providers [34]. One way to facilitate men’s participation in PCa screening is to promote the uptake of DRE and PSA tests and to emphasize that early detection and treatment may improve the prognosis of the disease [35]. The Prostate Cancer Advocacy Training had an important influence on the participants’ awareness and knowledge levels regarding PCa, as well as advocacy intentions as evidenced by their intent to share the information, they learned with their networks during the next 3 months following training.

Our study had several strengths. First, study participants were from two minority populations that experience disparities in PCa screening, including African American men who suffer the highest burden of PCa in the country. Second, training was supported by the use of educational materials developed by our team, tailored to the communities we serve. Third, training included presentations by concordant minority PCa scientists and clinicians, with whom participants could identify, with Hispanic/Latino/a clinicians and scientist delivering presentations in Spanish. Fourth, the use of simultaneous training of African American and Hispanic/Latino/a advocates from coast to coast in English and Spanish permitted bi-directional interactions. We acknowledge that our study also had several limitations; chief among them, the modest sample size, especially the small attendance at the Florida site, potentially limits generalizability and not particularly power since significant differences were observed. Future trainings with a larger sample, conducted in a similar manner will enable validation and refinement of the training/workshop program. Second, we encountered a limitation pertaining to the wording of posttest items 1–11 as shown in Table 5. Those items end with the words, “within the next 3 months” or “within the next month”; however, the pretest survey items excluded these words. This discrepancy could have introduced some errors, potentially towards the null if the pretest answer indicated intention to advocate anytime in the future, whereas the posttest response was restricted to the near future only. Additionally, data regarding participant “age” were not collected; thus, we cannot comment on potential influence by age group. Lastly, the lack of accurate responses about the age for PSA screening indicates that it may have not been well understood and that better describing of the screening age in our educational materials is warranted.

In summary, we report findings from an innovative Prostate Cancer Advocacy Training Workshop, developed specifically for African American and Hispanic/Latino men, that showed improvements in knowledge, awareness, and intention to engage in advocacy regarding PCa in the next 3 months after training completion. Larger sample sizes in the future will enable a more precise assessment of the knowledge progress. The study outcomes highlight the importance of developing culturally sensitive educational materials with the aims of imparting knowledge to community health workers and patient advocates and improving PCa knowledge and awareness among vulnerable populations and highlight important areas of knowledge deficiency that may benefit from wider implementation of this training in our communities, along with additional improvements to our training for further gains. Future studies are needed to assess if and how participants transfer this knowledge directly to their communities.
